# Analysis of genotype-by-environment interactions in a maize mapping population

**DOI:** 10.1093/g3journal/jkac013

**Published:** 2022-02-02

**Authors:** Asher I Hudson, Sarah G Odell, Pierre Dubreuil, Marie-Helene Tixier, Sebastien Praud, Daniel E Runcie, Jeffrey Ross-Ibarra

**Affiliations:** 1 Department of Evolution and Ecology, University of California, Davis, CA 95616, USA; 2 Center for Population Biology, University of California, Davis, CA 95616, USA; 3 Department of Plant Sciences, University of California, Davis, CA 95616, USA; 4 Center of Research of Chappes, Limagrain, Chappes 63720, France; 5 Genome Center, University of California, Davis, CA 95616, USA

**Keywords:** maize, multiparental populations, genotype ×, environment interactions

## Abstract

Genotype-by-environment interactions are a significant challenge for crop breeding as well as being important for understanding the genetic basis of environmental adaptation. In this study, we analyzed genotype-by-environment interactions in a maize multiparent advanced generation intercross population grown across 5 environments. We found that genotype-by-environment interactions contributed as much as genotypic effects to the variation in some agronomically important traits. To understand how genetic correlations between traits change across environments, we estimated the genetic variance–covariance matrix in each environment. Changes in genetic covariances between traits across environments were common, even among traits that show low genotype-by-environment variance. We also performed a genome-wide association study to identify markers associated with genotype-by-environment interactions but found only a small number of significantly associated markers, possibly due to the highly polygenic nature of genotype-by-environment interactions in this population.

## Introduction

Both the effect of a given genotype on a trait, and the impact of that effect on fitness, often vary across environments. Such genotype-by-environment interactions (G×E) are widespread, and have been commonly observed in plants (Bradshaw 1965; Des Marais et al. 2013). G×E interactions are of interest for multiple reasons: they provide insight into the physiological processes and genetic architecture underlying individual traits, are likely crucial for local adaptation of populations to different environments, but may also limit the response to selection (Allard and Bradshaw 1964; Kawecki and Ebert 2004).

While alleles affecting a trait will demonstrate G×E for fitness across environments when there is selection for different trait optima, it is also often observed that the effect of individual alleles on traits will vary as well. This indicates that these alleles affect plasticity and they may be present in a population due to selection for or against plasticity (Josephs 2018). Alternatively, they may be deleterious but rarely exposed to environments in which they are selected against, or unassociated with fitness and selectively neutral (Des Marais et al. 2013; Paaby and Rockman 2014).

One avenue to study G×E is to search for individual loci with changing effects on traits or fitness across environments. Multiple studies have identified loci that contribute to G×E [several of which are reviewed in Josephs (2018)]. Loci which contribute to G×E include the *EDA* locus in threespine stickleback fish, which is associated with adaptation to the freshwater environment, and *Sub1A* in rice, which is associated with tolerance to submergence (Xu et al. 2006; Barrett et al. 2008). Genome-wide association studies (GWAS) have also been used to identify alleles significantly associated with G×E, including shade response and drought response in *Arabidopsis thaliana* (Filiault and Maloof 2012; El-Soda et al. 2015).

Individual traits do not exist in a vacuum, however, and alleles that affect 1 trait often have pleiotropic effects on others. Indeed, the outcome of selection on a trait depends crucially on the genetic variance–covariance matrix (G-matrix), which describes how the genetic value at 1 trait covaries with genetic values at other traits (Lande 1979). Genetic covariation between traits can have profound impacts on the genetic response to selection, either hindering or facilitating trait response. For example, if fitness positively covaries with 2 different traits, but those traits negatively covary with each other, this can lead to a tradeoff.

But the G-matrix itself is not constant, as G×E at underlying loci may impact trait variation and covariation among traits (Wood and Brodie 2015). If in a different environment the covariance of a trait with fitness or other traits is weakened or changes sign, it may indicate that the selection or tradeoff does not exist in the new environment (Sgrò and Hoffmann 2004). As G×E contributes to the G-matrix within each environment, understanding the G-matrix in multiple environments may illuminate the causes of G×E. If the genetic covariance between 2 traits changes between environments and G×E is observed, then a change in the pleiotropy of the underlying loci may be responsible for both the changes in the genetic covariance and G×E.

Maize is a crop species adapted to a wide diversity of environments, from temperate to tropical and from low to high altitude (Hake and Ross-Ibarra 2015). G×E has been shown to be an important contributor to many traits in maize, including grain yield (Gage et al. 2017; Gates et al. 2019; Rogers et al. 2021). Nonetheless, identification of G×E in maize, as in many species, is complicated by issues of population structure and the low minor allele frequency of most polymorphisms (Korte and Farlow 2013). To circumvent these issues, we investigated the genetic basis of G×E in maize in a multiparent advanced generation intercross (MAGIC) population of 16 diverse temperate maize lines (Odell et al. 2022). We grew the MAGIC hybrids across 5 contrasting temperate environments with diverse management practices in order to capture a broad range of G×E relevant to the conditions the parental lines would be grown in.

We find that G×E contributes as much as genotypic main effects to variance for some traits. While G×E interactions are significant, genome-wide association only finds a small number of markers significantly associated with G×E interactions, perhaps reflecting the highly polygenic nature of most traits. Nonetheless, estimation of the G-matrix in each environment reveals that changes in genetic covariance are common and may be contributing to observed G×E. For example, we find that while only a small proportion of variance in flowering time depends on G×E, the genetic covariance between flowering time and grain yield is strongly affected by the environment.

## Materials and methods

### Plant materials

We developed a maize MAGIC population by repeatedly crossing the offspring of 16 maize inbred lines to generate recombinant individuals (Odell et al. 2022). Inbred lines were selected to maximize genetic diversity and include dent, flint, and European flint lines. After 8 generations of intercrossing, we generated a population of 344 doubled haploids (DHs) lines. DH lines were crossed to MBS847, a dent line chosen to be the tester, to make F1 plants.

### Phenotype data

The MAGIC F1 plants were phenotyped in 4 different field locations in 4 different years, resulting in 5 distinct environment-years (Supplementary Fig. 1 and Supplementary Table 1). The environment-years included Blois, France, in 2014 and 2017, Nerac, France, in 2016, St. Paul, France, in 2017, and Graneros, Chile, in 2015. We used an alpha design with 2 plots of around 80 plants grown for each genotype in each environment-year. Planting density ranged between 85,000 and 95,000 seeds per hectare. Seeds were planted with an automatic seed drill. The row width was 0.8 meters with 2 rows per plot. The fields in environment-years Blois 2014, Blois 2017, and Graneros 2015 all received consistent irrigation. The field in Nerac 2016 was not actively irrigated from vegetative phase through flowering, causing drought stress through most of the life cycle. The field in St. Paul 2017 was not irrigated during vegetative phase but was irrigated during flowering to allow plants to recover from the earlier drought stress. The applied drought stress was mild and intended to be representative of realistic field conditions.

We measured the following traits: male flowering date, female flowering date, anthesis-silking interval (ASI), plant height, % harvest grain moisture (HGM), grain yield, and thousand kernel weight (TKW), where values were averaged over plots. Both flowering time phenotypes were measured as the sum of degree days since sowing with a base temperature of 6°C (48°F). Male flowering date was considered as the growing degree days (GDD) until 50% of plants in a plot were shedding pollen on approximately 1 quarter of the central tassel spike. Female flowering date was considered as the GDD until 50% of plants in a plot were flowering with 2 cm of silk outside of husk leaves. Plant height was measured as the distance from the base of the plant to the top of the tassel. Grain was collected using a combine harvest. Grain yield and TKW were both adjusted to 15% humidity. TKW was estimated from a 100 kernel sample. Data was also collected from an additional environment, Szeged, Hungary in 2017. We did not use this data in the analyses presented here as flowering date was not collected on the same schedule as in the other environments and this caused issues with the G×E analyses. Data from Szeged are available in the data repository associated with this paper. Between 292 and 309 of the MAGIC F1 lines were grown in each environment. There were a total of 325 lines that had both genotype data and phenotype data from at least 1 environment.

### Genotyping

We genotyped each of the DH lines using the Affymetrix Axiom Maize Genotyping Array, which successfully genotyped 551,460 SNPs. The probability of each founder contributing to each segment in the genome was imputed from the genotyped SNPs (Odell et al. 2022).

### Estimating kinship

Kinship matrices for the DH lines were estimated from the genotyped SNPs using the VanRaden method as implemented in the R package *sommer* (Covarrubias-Pazaran 2016; VanRaden 2008; R Core Team 2020). SNPs were first filtered for linkage disequilibrium using Plink with a window size of 50 kb, a step size of 5, and an *r*^2^ threshold of 0.2 (Purcell et al. 2007). In order to perform genome-wide association analyses, we used the leave 1 chromosome out method (Lippert et al. 2011).

### Genotype × environment interactions

Variance components for each trait were estimated using the R package *sommer*. We used the formula:

$$y=Z_{G}u_{G}+Z_{E}u_{E}+Z_{E:G}u_{E:G}+f_{E}(x,y)+e,$$

where **y** is a vector of *n* observations from individual plots of a single trait including both plots of all lines in all environments, $y=Z_{G}u_{G}+Z_{E}u_{E}+Z_{E:G}u_{E:G}+f_{E}(x,y)+e$ is a *n* × *r* design matrix for the genotypic main effects of the *r* lines, $Z_{E}$ is a n×5 design matrix for the environmental main effect, $Z_{E:G}$ is a n×5r design matrix for genotype-specific effects in each environment, $u_{G}$ is a length *r* vector of random genotypic effects, $u_{E}$ is a length 5 vector of environmental random effects, $u_{E:G}$ is a length 5*r* vector of random G×E effects with same variance and covariance among environments, $f_{E}(x,y)$ is a 2-dimensional spline for the effect of the *x/y* position in the field nested within environment modeled as a single random effect fit from an incidence matrix containing the tensor products of the *x* and *y* coordinates in the field, and **e** is the error. *sommer* models 2D splines based on modified code from *SpATS* (Rodríguez-Álvarez et al. 2018).

### Genome-wide association studies

Genome-wide association analyses for loci contributing to G×E interactions were performed with the R package *GridLMM* (Runcie and Crawford 2019). Imputed founder probabilities at each locus were used as markers, meaning that at each marker we asked if the identity of the founder which contributed that genomic region at a given locus was a significant predictor of differences in plasticity among the hybrids. We set GridLMM to obtain maximum likelihood estimates of the effect of each marker.

G×E models can be parameterized in multiple ways which could potentially capture different aspects of G×E. We chose to model G×E in 3 different ways in our GWAS analyses, which we describe below.

#### Main effect across environments and deviation effect within environments

We tested whether a locus had a different effect on a trait in 2 environments: Blois 2017 and Nerac 2016. We chose these 2 environments because they were respectively the highest and lowest yielding environments. The model for this GWA was:

$$\begin{array}{c} y=\mu +w\alpha +X_{m}\beta_{m}+X_{E:m}\beta_{E:m}+Z_{G1}u_{G1}+Z_{E:G1}u_{E:G1}\\ +Z_{G2}u_{G2}+e, \end{array}$$

where **y** is a vector of *n* observations from individual plots of a single trait including both plots of all lines in both environments, μ is a constant length *n* vector of the average trait value across the 2 environments, **w** is a length *n* design matrix of environmental effects taking values of −1 and +1 according to the environment (1 for Blois 2017 and −1 for Nerac 2016), *α* is a scalar representing ½ the deviation of trait means between the 2 environments, $X_{m}$ is a n×16 matrix, where the *k*th column is the probability that each of the *n* individuals inherited from the *k*th founder at marker *m*, $X_{E:m}$ is an *n* × 16 matrix formed by multiplying **w** with each column of $X_{m},\;\beta_{m}$ is a vector of main effects of the founder alleles averaged over the 2 environments, $\beta_{E:m}$ is a vector of differences between the founder allele effects between the 2 environments, $Z_{G1}$ is a *n* × *r* design matrix of additive genotypic effects, $Z_{E:G1}$ is a *n* × *r* design matrix of genotype deviations formed by multiplying each column of $Z_{G1}$ by **w**, $Z_{G2}$ is a *n* × *r* design matrix of nonadditive genotypic effects, $u_{G1}$ is a vector of additive genotypic effects averaged over the 2 environments, $u_{E:G1}$ is a vector of additive genotypic deviations between the 2 environments, $u_{G2}$ is a vector of nonadditive genotypic effects averaged across the 2 environments, and **e** is a vector of error terms. $u_{G1}$Z$u_{G1}$ and $u_{E:G1}$ both have covariance proportional to **K**, where **K** is the additive genetic relatedness matrix, and $u_{G2}$ and **e** both have covariance proportional to the identity matrix. The statistical test to identify markers influencing G×E was against H0: $\beta_{E:m}=0$.

#### Plasticity

We tested whether a locus had an effect on the slope of the observations of a genotype across the mean phenotypic value of all genotypes in an environment. This model has the benefit of including the maximum amount of data. Compared to the main effect and deviation model (1), this model might be more likely to pick up G×E effects that have smaller effects within those 2 environments but a larger effect on the overall slope across environments. The model is the same as in (1) except for the following: we now include all 5 environments, **w** is a length *n* vector with each element taking the mean value of the phenotype within the environment of the observation, and μ is a length *n* vector of the mean value of the phenotype within the environment of the observation.

#### Finlay–Wilkinson GWAS

Finally, we tested whether a locus had an effect on the slope of the observations of a genotype across the mean grain yield of all genotypes in an environment. Mean grain yield here serves as a proxy for stress or environment quality and as such this GWA is testing whether a locus affects the response to stress. This is known as a Finlay–Wilkinson analysis (Finlay and Wilkinson 1963). For this analysis, a quantile plot of *P*-values indicated that the test was poorly calibrated. Instead of asking whether allowing a marker to have a slope across environments improved prediction of a trait in each environment as in (2), we thus asked whether the marker significantly predicted the slope of each genotype.

$$s=X_{m}\beta_{s}+Z_{G1}u_{s}+e,$$

where **s** is a length *r* vector of slopes for each genotype of trait values on mean grain yield in each environment, $\beta_{s}$ is a vector of marker effects, and $u_{s}$ is a vector of genotypic effects with covariance proportional to **K**. Other model terms are as in (1).

To determine significance thresholds for the first 2 models, we permuted phenotypic values among lines within each environment and ran the GWA 100 times. For the third model, we permuted the slopes among the genotypes and ran the GWA 100 times.

### The G-matrix across environments

We estimated the G-matrix in each environment using the R package *brms* (Bürkner 2017). *brms* implements Bayesian multilevel models using Markov chain Monte Carlo (MCMC) algorithms. This is important as the samples from the MCMC chains allow us to estimate uncertainty and significance in our downstream analyses. We used the model:

Y=ZU+f(x,y)+E,

where $Y=[y_{1}\ldots y_{5}]$ and $y_{i}$ is a vector of *n* observations for the *i*th trait, **Z** is a *n* × *r* design matrix of genotypes, **U** and **E** are random effects drawn from multivariate normal distributions: $\mathrm{vec}(U)∼N(\mathrm{vec}(0),G⊗I_{r}),\;\mathrm{vec}(E)∼N(\mathrm{vec}(0),R⊗I_{n}),\;I_{r}$ is the *r* × *r* identity matrix where *r* is the number of lines grown in an environment, $I_{n}$ is the *n* × *n* identity matrix where *n* is the number of observations, and **G** and **R** are 5 × 5 genetic variance–covariance and residual variance–covariance matrices estimated from the data. *G* and *R* are parameterized as the products of standard deviations and correlation matrices with a half Student-*T* distribution and LKJ-correlation prior. f(x,y) is a 2-dimensional spline for the effect of the x/y position in the field. The standard deviations of the 2 splines have half Student-*T* distributions as priors.

All traits were scaled by the mean value across all environments and centered before analysis in order to make them unitless and improve model convergence. We performed this same analysis with nonscaled traits so that our results can be compared with those of previous studies with nonscaled phenotypic data. The G-matrices we estimated were broad sense G-matrices as they included both additive and nonadditive sources of genetic variance. We ran 4 chains with 1,500 iterations of burn-in followed by 3,500 iterations. We chose these numbers as the *brms* documentation states that most models will converge with only a few thousand iterations. We assessed convergence by checking that all statistics output by *brms*—such as $\hat{R}$, defined as the potential scale reduction factor on split chains, and the number of divergent transitions, which occur when the simulated trajectory along the posterior differs from the true trajectory—were within recommended ranges and by visually inspecting the trace and autocorrelation of model parameters. For genotypic standard deviations and correlations, the bulk effective sample size of parameters ranged from 1,506 to 6,449. To determine whether the correlation between 2 traits differed between environments, we found the difference between the MCMC samples for the 2 environments and determined whether the interval spanned by the 2.5% and 97.5% quantiles of the differences overlapped zero. In particular, if the correlation between 2 traits was positive in 1 environment and negative in another, and if one or both of those traits correlate with yield, this would be evidence for a possible tradeoff between fitness in different environments.

To quantitatively assess differences among the G-matrices estimated in the 5 environments, we performed eigenanalysis of a covariance tensor as described in Aguirre et al. (2014). The tensor approach is a geometric approach founded on the diagonalization of symmetric matrices, and is mainly used to calculate a set of orthogonal axes known as eigentensors that describe coordinated changes in the elements of the original matrices being compared. Eigentensors describe which elements of a set of matrices most contribute to variation among those matrices. As the G-matrices differed in their environment but not population, the genetic variances and covariances that contribute the most to the eigentensors are those which are most influenced by the environment. Eigentensor analysis was performed on the posterior median G-matrices. Uncertainty in the eigentensors was estimated by performing eigentensor analysis on the MCMC samples of the G-matrices. Finally, to determine whether an eigentensor explained more of the variation among G-matrices than would be expected by chance, we shuffled the real phenotypic data among environments, estimated G-matrices, and asked whether the eigentensors of the randomized G-matrices explained as much of the variation as the MCMC samples from the real data. If an eigentensor of the estimated G-matrices explain more of the variation, this indicates that this eigentensor is explaining biological variation and not only variation due to random sampling.

## Results

We evaluated 7 phenotypes for each of 344 hybrids of DH lines crossed with a tester in replicated trials across 5 environments that varied in temperature, daylength, and watering or drought conditions (Supplementary Fig. 2). Each DH line hybrid was genotyped for 551,460 SNPs, allowing us to identify ancestry segments along the genome.

### Genotype × environment interactions

**Fig. 1.**
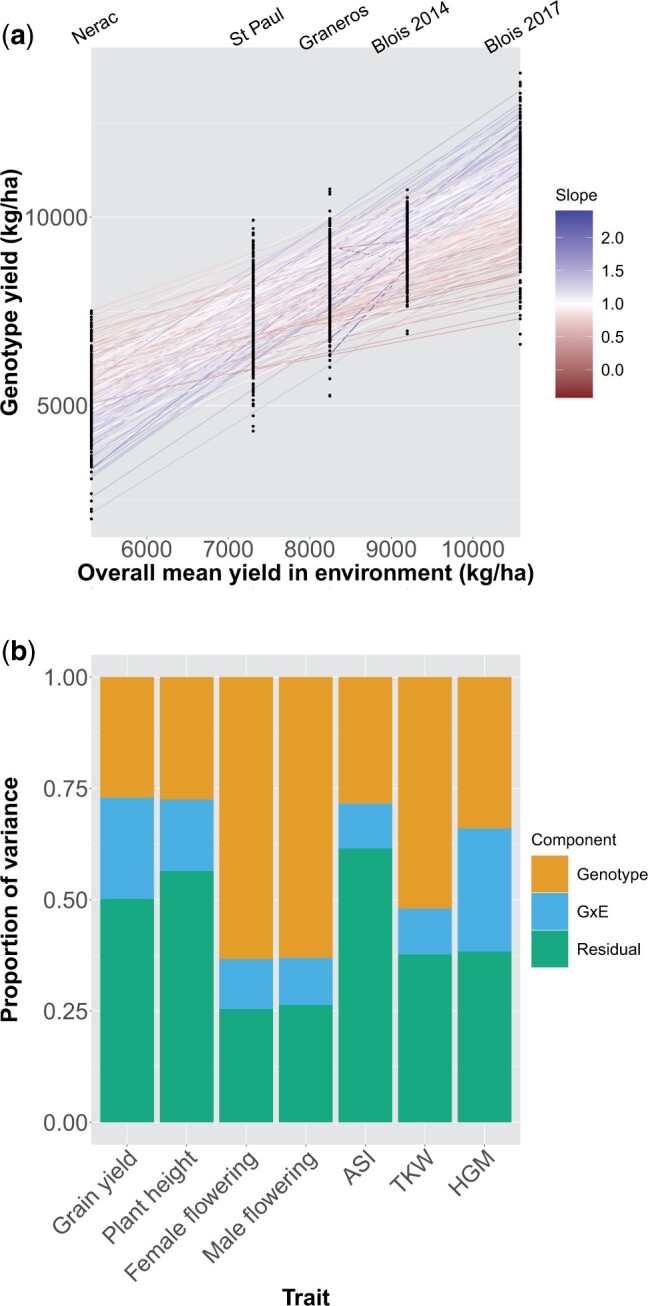
a) Mean yield of all genotypes in each environment. On the *x*-axis environments are plotted by the mean yield across all genotypes in that environment. Points are mean yields of individual genotypes. Lines are the slope of a genotype’s mean yield in each environment on the mean yield of all genotypes in that environment. The color of the line corresponds to the slope; a slope greater (or less) than one indicates a genotype more (or less) responsive to the environment than average. b) Restricted maximum likelihood estimates of variance components for each trait across all environments.

Genotypic main effects and G×E interactions contributed a significant amount of the variance of all measured traits (Fig. 1). Across environments, it was common for the rank of DH lines for grain yield to change, indicating that individual lines were generally not high yielding in all conditions (Fig. 1a). ASI showed a qualitatively similar pattern of rank-changing, while some traits such as TKW showed less dramatic G×E (Supplementary Fig. 3). The proportion of variance due to main genotypic effects ranged from 0.34 for grain yield to 0.72 for male flowering date (Fig. 1b). For grain yield and HGM, G×E interactions contributed an amount of variance similar to the amount contributed by genotypic effects. For flowering time, TKW, and plant height, G×E interactions contributed less of the variance than main genotypic effects.

### Genome-wide association studies

**Fig. 2.**
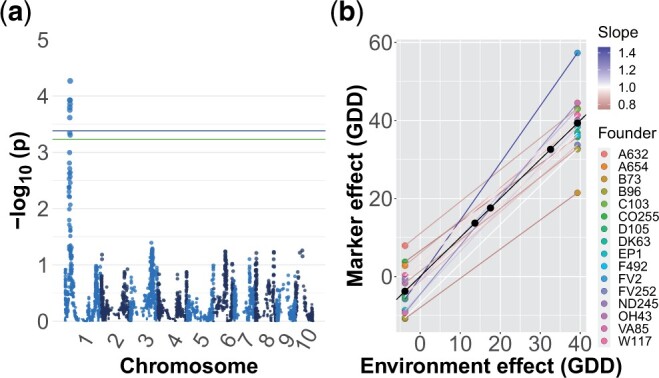
a) Manhattan plot for plasticity (model ii) GWAS on ASI. The blue and green lines represent the 5% and 10% significance levels based on permutation tests, respectively. b) Estimated effect of founder ancestry on plasticity for the most significant marker. The slope of a line indicates the plasticity of that haplotype and the difference in slopes is G×E. The color of the line corresponds to the slope; a slope greater (or less) than one indicates a genotype more (or less) responsive to the environment than average.

Our test of the deviation effect of a marker within environments did not recover any markers significant at the 5% permutation threshold for any trait. In contrast, our plasticity GWAS identified 2 peaks which were significant at the 5% significance level, which were for ASI and female flowering (Fig. 2a and Supplementary Fig. 4). Neither of these peaks overlapped with GWAS peaks for main effects in this population (Odell et al. 2022). The peak for ASI on chromosome 1 appears to be driven by the effect of the FV2 founder, which has a small effect in environments where ASI is close to zero but strongly increases the magnitude of ASI in environments where average ASI is greater (Fig. 2b). Patterns of identity by descent at the genomic region surrounding the peak identified unique haplotypes for 15 of the founders (Odell et al. 2022), but a PCA of the SNPs in the region did not indicate that the FV2 haplotype was strongly diverged from other founders (Supplementary Fig. 4). The peak for female flowering on chromosome 4 appears to be driven by founder A654, but the marker effects for this founder appeared unrealistically strong and likely reflect an artifact of the extremely low sampling of this founder among the DH lines. In addition to these 2 associations at the 5% level, we detected 1 peak which was significant at the 10% level for grain yield (Supplementary Fig. 6). Our Finlay–Wilkinson GWAS uncovered 1 peak significant at the 5% level for ASI (Supplementary Fig. 7). However, the founder whose effect appears to be driving this peak also appears to be underrepresented at this locus and only 1 line has a greater than 0.8 probability of carrying this founder allele. As a result, this peak is likely to be a statistical artifact.

### The G-matrix across environments

**Fig. 3.**
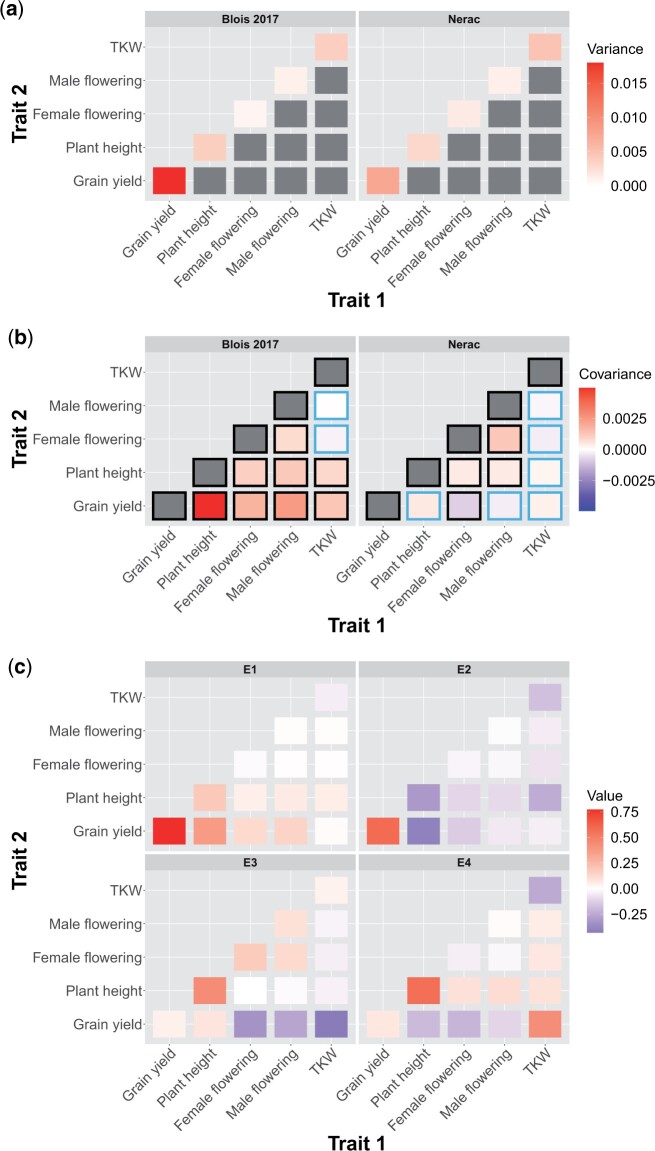
The genetic a) variances and b) covariances of the highest yielding environment (Blois 2017) and the lowest yielding environment (Nerac 2016). Traits are mean scaled. A black border around a covariance indicates that the 95% quantile interval of the posterior does not overlap with zero. Note that the scales on the upper and lower rows are different. c) Contributions of elements in the genetic variance–covariance matrices to the first 4 eigentensors of the set of genetic variance–covariance matrices. Elements on the diagonal are genetic variances of traits and elements on the off-diagonals are genetic covariances between traits. The color of a square represents the strength of the contribution of that element to the eigentensor, which is not dependent on the sign.

To understand how the environment affected pleiotropy, we estimated the genetic variance/covariance matrix (G-matrix) of 5 traits in each environment (Fig. 3, a and b and Supplementary Figs. 8 and 9). We dropped ASI and HGM from this analysis because models including those traits failed to converge; ASI was dropped due to concerns about collinearity as it is a function of 2 other traits in our analysis and HGM was dropped because in analyses run on subsets of these traits we found that HGM had very low covariance with the other traits. Comparisons of the 95% credible intervals of the difference between individual genetic correlations revealed numerous differences among environments (Supplementary Fig. 10). Both the genetic variances of individual traits and the covariances between traits differed across environments (Fig. 3, a and b). As the traits were mean scaled, the variances presented in Fig. 3a are not heritabilites, which is the genetic variance scaled by the phenotypic variance. Importantly, mean-scaled genetic variances are not affected by the amount of residual variance, which means that a trait with high genetic variance relative to the mean along with high environmental variance can have low heritability but high mean-scaled genetic variance. (Houle 1992). We found that grain yield generally had high mean-scaled genetic variance in each environment, and the single highest mean-scaled genetic variance of any trait in any environment was grain yield in Blois 2017. In 1 case, the sign of a genetic covariance changed: the genetic covariance between grain yield and female flowering date was positive across all environments except in Nerac 2016. This environment was the only 1 in which the values in the 2.5% and 97.5% quantiles of the posterior of the genetic covariance between grain yield and female flowering date was entirely negative, while in both years in Blois this interval was positive. The median posterior values of some other genetic covariances also switched signs between environments, but based on credible intervals we cannot state that they switched with confidence.

To quantitatively assess how individual elements of the G-matrix contributed to variation among environments, we performed an eigentensor analysis. The eigentensors of a set of G-matrices describe independent dimensions of variation among the G-matrices and can be used to identify which elements are contributing the most variation among the set. All of the 4 nonzero eigentensors explained significantly more variance than expected by chance (Supplementary Fig. 11). The element of the G-matrix that most contributed to the first eigentensor was genetic variance for grain yield (Fig. 3c). When plotting each environment on this eigentensor, Blois 2017 is strongly differentiated from the other environments, which is probably due to the genetic variance for grain yield being the highest in this environment (Supplementary Fig. 12). The genetic variance for grain yield also contributed strongly to the second eigentensor, while the genetic covariance between plant height and grain yield and the genetic variance of plant height contributed in the opposite direction. The third eigentensor described a contrast between genetic variance for plant height on the one hand and the genetic covariances between both female flowering date and TKW with grain yield on the other. Nerac is strongly differentiated on this eigentensor. While the covariance between female flowering and grain yield is not the only element of the G-matrix contributing to the third eigentensor, it is worth noting that Nerac is the only environment in which this covariance is negative.

Results of the analysis with nonscaled phenotypes are presented in the Supplementary figures.

## Discussion

### Genotype × environment interactions

Genotype × environment interactions are known to be important for many agronomically important traits in maize, and our results on the relative importance of G×E across traits confirm these earlier findings. For example, male and female flowering date have been shown to be influenced predominantly by additive genetic effects and are not strongly influenced by G×E interactions (Buckler et al. 2009; Rogers et al. 2021), while grain yield and HGM have large G×E variance components relative to main genotype effects (Gage et al. 2017; Rogers et al. 2021). We find similar results in our analysis, indicating that this may be a consistent pattern for diverse maize germplasm in temperate environments.

If genotypes are adapted to different environments, we would expect to see G×E for fitness-related traits. The high variance contributed by G×E to grain yield seen in this study thus indicates that the founder maize lines, despite all having been bred in temperate environments, still carry many alleles that are differentially adapted to this set of environments. For traits that are further removed from fitness it is less clear how to interpret the contribution of G×E. It may be that the G×E we observe for a trait like HGM, which has a high proportion of G×E variance and a low genetic covariance with grain yield, is an example of neutral plasticity and is not under strong selection (Des Marais et al. 2013).

### Genome-wide association studies

Despite the presence of substantial G×E variance for several traits, we found relatively few markers which were significantly associated with G×E. One possible explanation is that the G×E variance we observed is largely polygenic and caused by many loci of small effect which we did not have power to detect with our GWAS. Previous studies investigating loci with main effects on traits such as grain yield and flowering time in maize have found that they are highly polygenic (Buckler et al. 2009; Dell’Acqua et al. 2015). It may not be surprising then if G×E for these traits also has a similarly polygenic basis. Grain yield is a highly integrated trait dependent on the interaction of many other traits with the environment; if those traits have a complex basis and different optima within different environments, then it would not be surprising to observe large G×E variance at the level of genotype while not observing significant G×E effects for individual loci.

### The G-matrix across environments

The G-matrix has previously been shown to differ as much between environments as between populations (evidence reviewed in Wood and Brodie 2015). Our work shows that the G-matrix differs across environments in a multiparent population of temperate maize lines. We find that these differences include both changes in the magnitude of genetic variances and covariances as well as changes in the sign of genetic covariances. The highest mean-scaled genetic variance we observed was for grain yield in Blois 2017, and in general grain yield had high mean-scaled genetic variance compared to other traits within each environment. This is in contrast to the finding that grain yield had the lowest heritability across all environments. This finding fits with previous work finding that fitness proximal traits frequently have low heritability but high mean-scaled genetic variance, possibly because of high residual variance for fitness proximal traits reducing heritability (Houle 1992).

The magnitude of the genetic covariances between traits can be reduced solely as a function of reduced genetic variance for one or both of these traits without a change in the correlation between them. However, by looking at genetic correlations, we show that the correlations between traits varied across environments beyond effects of the differences in the variances (Supplementary Fig. 10). In addition, changes in the genetic variance alone will not cause the covariance between traits to change sign, which we also see for some combinations of traits. Particularly striking was the change in sign for the genetic covariance between grain yield and female flowering date observed in the most stressful environment, Nerac 2016. This environment was the only 1in which the genetic covariance between grain yield and female flowering date was negative. Previous work has shown that flowering time is important for adaptation to drought stress (reviewed in Kazan and Lyons 2016). Nerac 2016 experienced a drought from vegetative growth through maturity. Early flowering in this environment was genetically correlated with higher yields, suggesting that early flowering may have been a means to escape drought stress. The change in sign of the covariance is noteworthy given that we observed low G×E variance and high genotypic variance for female flowering date while simultaneously observing high levels of G×E variance for grain yield. This indicates that genotypes were relatively consistent in their flowering time across environments but that late flowering genotypes were higher yielding in most environments and lower yielding in 1 environment. In this way, a change in the genetic covariance between 2 traits (grain yield and female flowering) across environments may be contributing to G×E in one of those traits (grain yield), and provides an illustrative example of how traits that themselves show little G×E may nonetheless contribute to G×E for fitness.

While differences between environments presumably shape these changes in the G-matrix, previous work has found that neither measures of environmental novelty nor differences in phenotypic means predicted differences in the G-matrix when looking across all the studies in a meta-analysis (Wood and Brodie 2015). In our analysis we find a similar result; differences between the G-matrices estimated in each environment are largely idiosyncratic and do not correspond with levels of stress or water availability. Eigentensor analysis reveals that each of the main directions of variation across G-matrices correspond mostly to the differentiation of one or at most two of the environmental G-matrices from the others. Previous work investigating the G-matrix of plant populations grown in well-watered and drought environments has been inconsistent in terms of whether drought stress increases or decreases genetic variance and how it affects the genetic correlation between flowering time and yield (Sherrard et al. 2009; Manzaneda et al. 2015). Considering our work in the context of previous studies, we suggest that the environmental contribution to the G-matrix is complex and not easily described by 1 environmental axis, which raises the possibility that multivariate adaptation to the environment may be difficult to predict.

In addition, both the severity and timing of drought seem to be important in determining the effects of water deficit on covariances between traits. In this study, we find that in Nerac, the most drought-stressed environment, the genetic covariance between flowering time and yield is negative and that this genetic covariance contributes to differentiating it from the other environments. The fact that the genetic covariance between flowering date and grain yield in the other water deficit environment, St. Paul, was not significantly negative may be because that population was given water during flowering while in Nerac water deficit extended through flowering. It appears that how the G-matrix is affected by environmental stress is highly dependent on the species and population studied and the exact stress applied.

### Conclusion

Using a MAGIC population of maize grown in 5 environments × year combinations we were able to analyze the genetic basis of G×E in a set of diverse maize lines. We observed G×E variance for all traits and for some traits we observed comparable amounts of genotypic and G×E variance. Estimating the G-matrix within each environment revealed that changes in genetic variances and covariances across environments were common. Notably, the genetic covariance between yield and female flowering time was positive in most environments but negative in 1 of the environments. GWAS identified 1 locus significantly associated with G×E for ASI. Given the substantial G×E variance, the low number of significant loci suggests that G×E for the traits we analyzed may have a polygenic basis.

## Data availability

Phenotypic and environmental data are located on Figshare with doi 10.6084/m9.figshare.14963034. Genotypic data are available through a data repository associated with companion paper (Odell et al. 2022).

Supplemental material is available at *G3* online.

## Supplementary Material

jkac013_Supplementary_Data
