# Supplementary material for: Analysis of genotype-by-environment interactions in a maize mapping population
Source: G3 (Bethesda). 2022 Feb 2;12(3):jkac013. doi: 10.1093/g3journal/jkac013 (PMC8895993; doi:10.1093/g3journal/jkac013)
Supplement: jkac013_Supplementary_Data [file jkac013_supplementary_data.pdf]

SUPPLEMENTAL FIGURES

Table S1 Features of the five growing environments.

Environment-Year	Mean temperature (℃)	Mean relative humidity (%)	Mean precipitation (mm)	Water treatment[a]	Planting density (seeds/hectare)
Blois 2014	16.7	75.2	2.19	OPT	85,000
Blois 2017	17.0	72.3	1.71	OPT	95,000
Graneros 2015	20.1	55.1	0.266	OPT	90,000
Nerac 2016	19.1	74.9	1.15	Early term	85,000
St. Paul 2017	20.3	65.4	1.12	Recovery	90,000

[a] OPT is optimum watering, Early Term is water deficit during vegetative growth through maturity, and Recovery is water deficit during vegetative growth with recovery at flowering time.

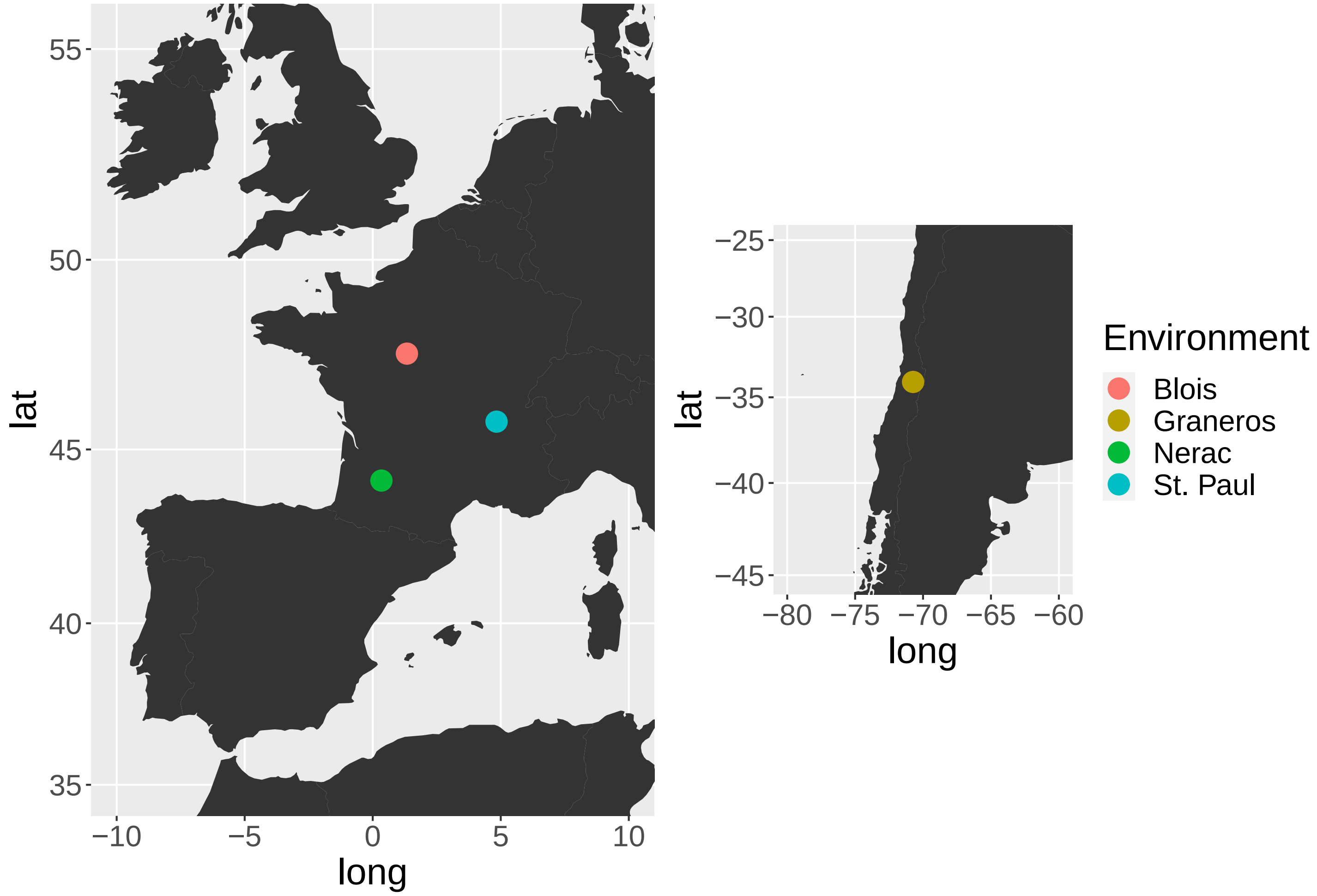

Figure S1 Locations of the environments the MAGIC population was grown in. In one environment (Blois) the MAGIC population was grown in two years.

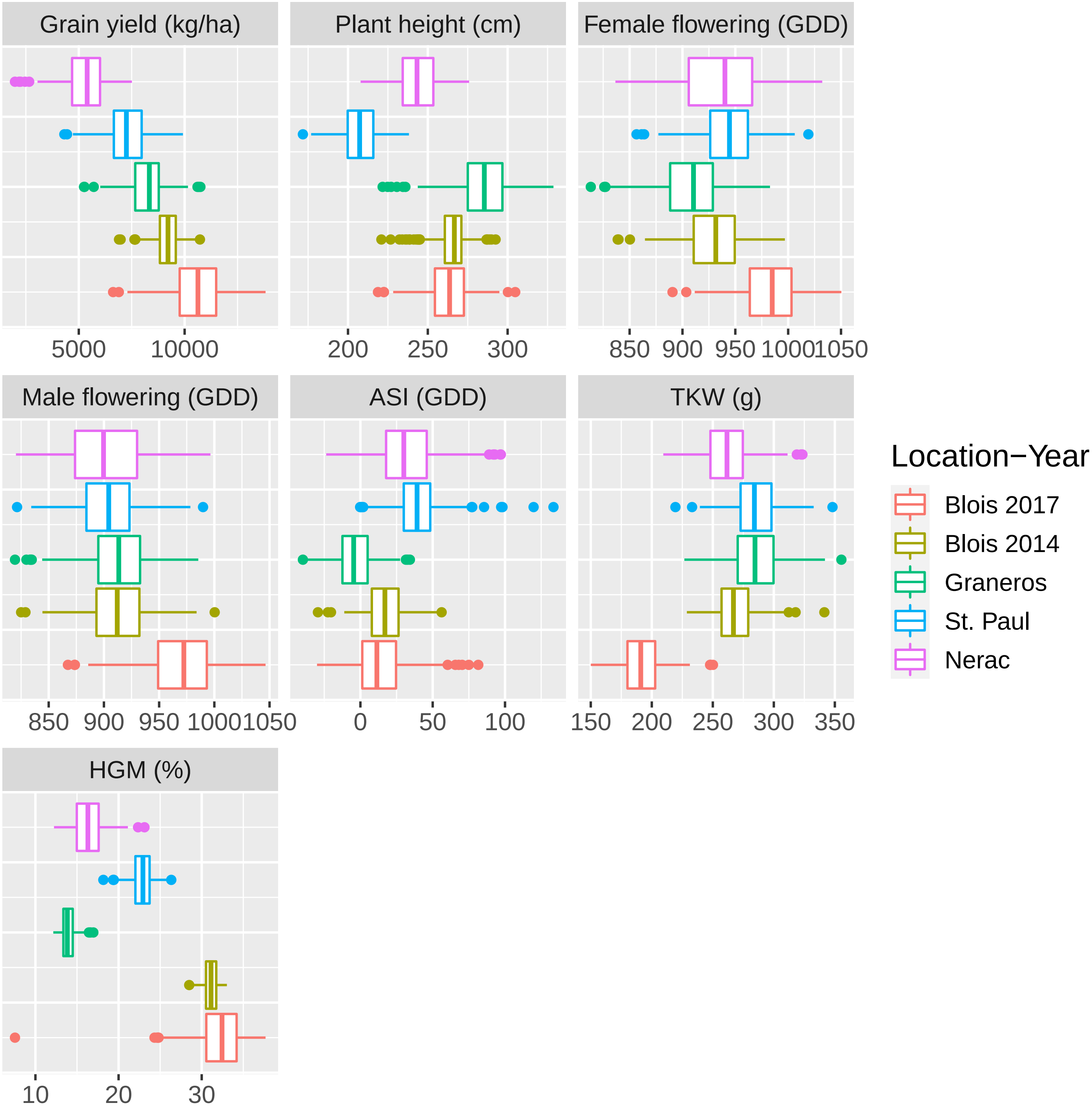

Figure S2 Phenotypes of the MAGIC population in each environment. Box plots show median as line in box, 25% and 75% quartiles as edges of box, and most extreme values that are at most 1.5 * inter-quartile range as whiskers, with any values more extreme shown as individual dots.

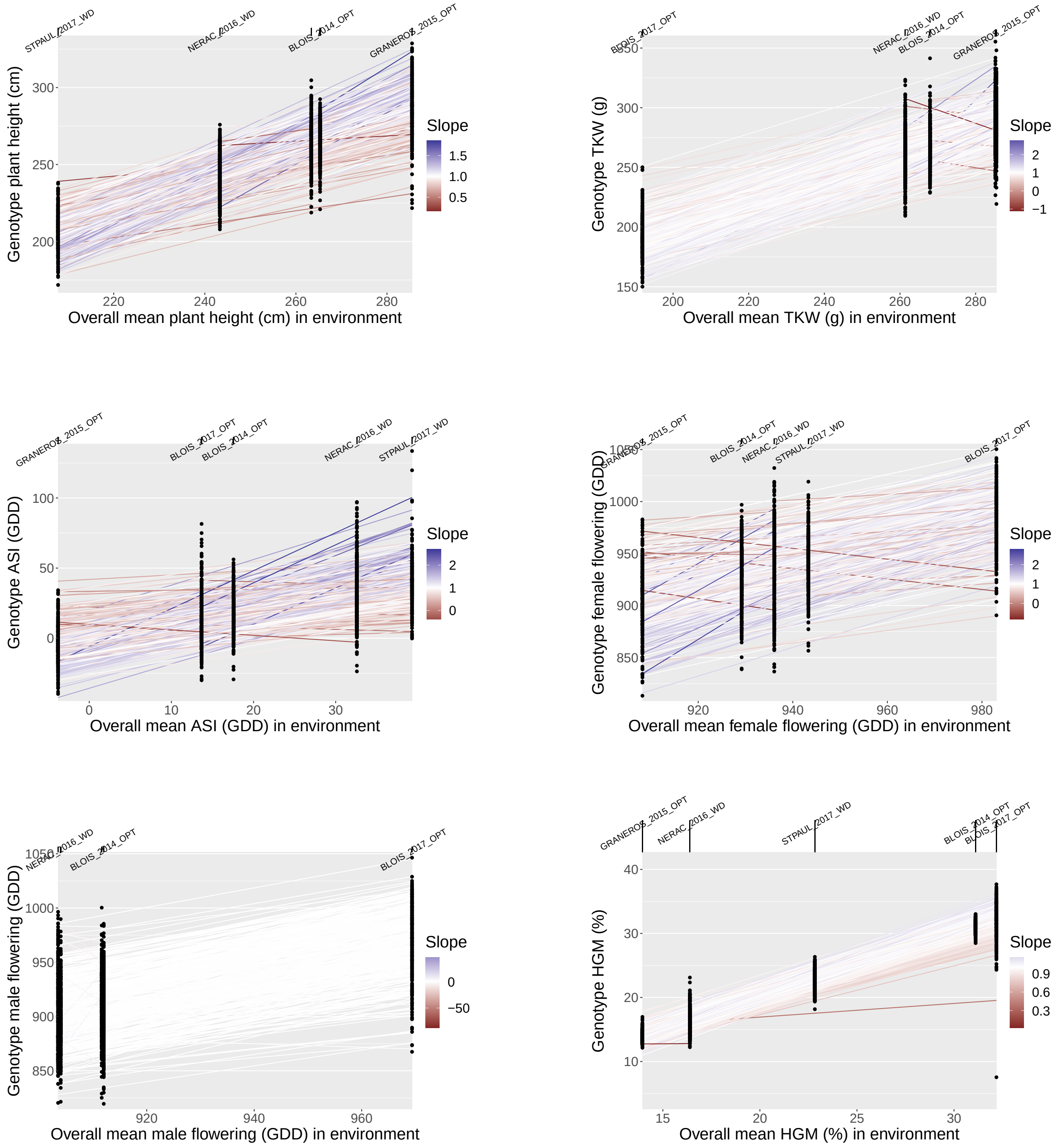

Figure S3 Mean trait values of all genotypes in each environment. On the X axis environments are plotted by the mean of each trait across all genotypes in that environment. Circles are the mean trait values of individual genotypes. Lines are the slope of a genotype's mean trait value in each environment on the mean trait value of all genotypes in that environment. The color of the line corresponds to the slope; a slope greater (or less) than one indicates a genotype more (or less) responsive to the environment than average.

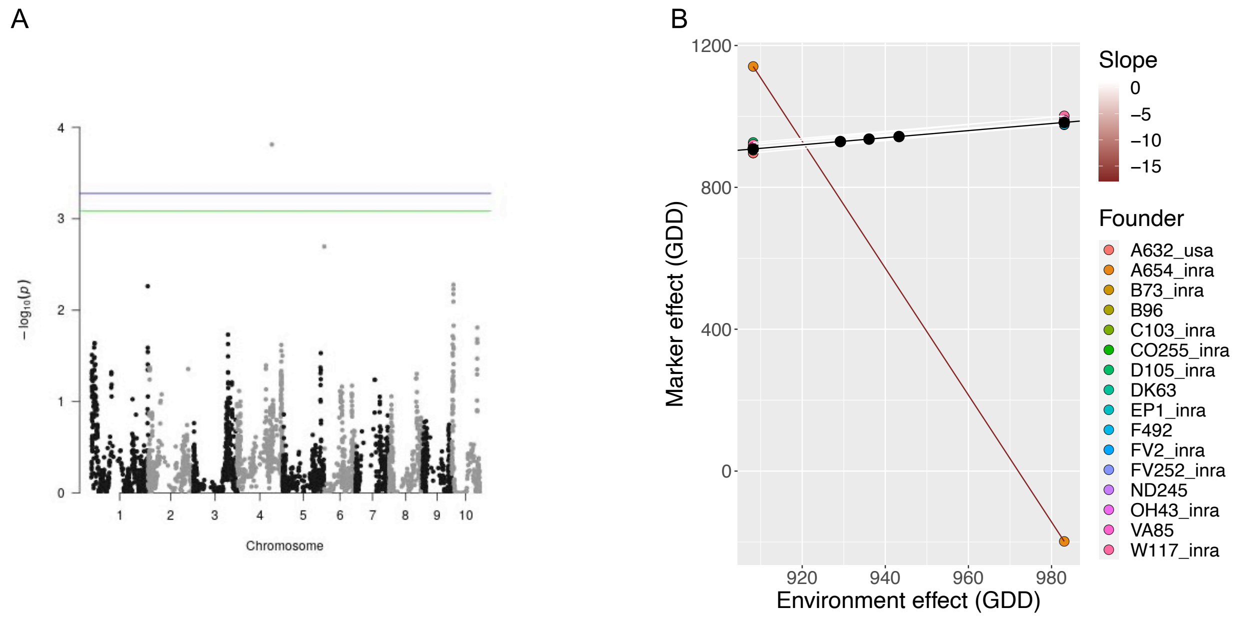

Figure S4 A) Manhattan plot for plasticity GWAS on female flowering. The blue and green lines represent the 5% and 10% significance levels based on permutation tests, respectively. B) Estimated effect of founder ancestry on plasticity for the most significant marker. Lines are the slope of a marker's effect in each environment on the mean female flowering date of all genotypes in that environment. The color of the line corresponds to the slope; a slope of one indicates a marker with the average response to the environment, a slope less than one indicates a marker less responsive to the environment than average, and a slope greater than one indicates a marker more responsive to the environment than average. Effect sizes in GDD.

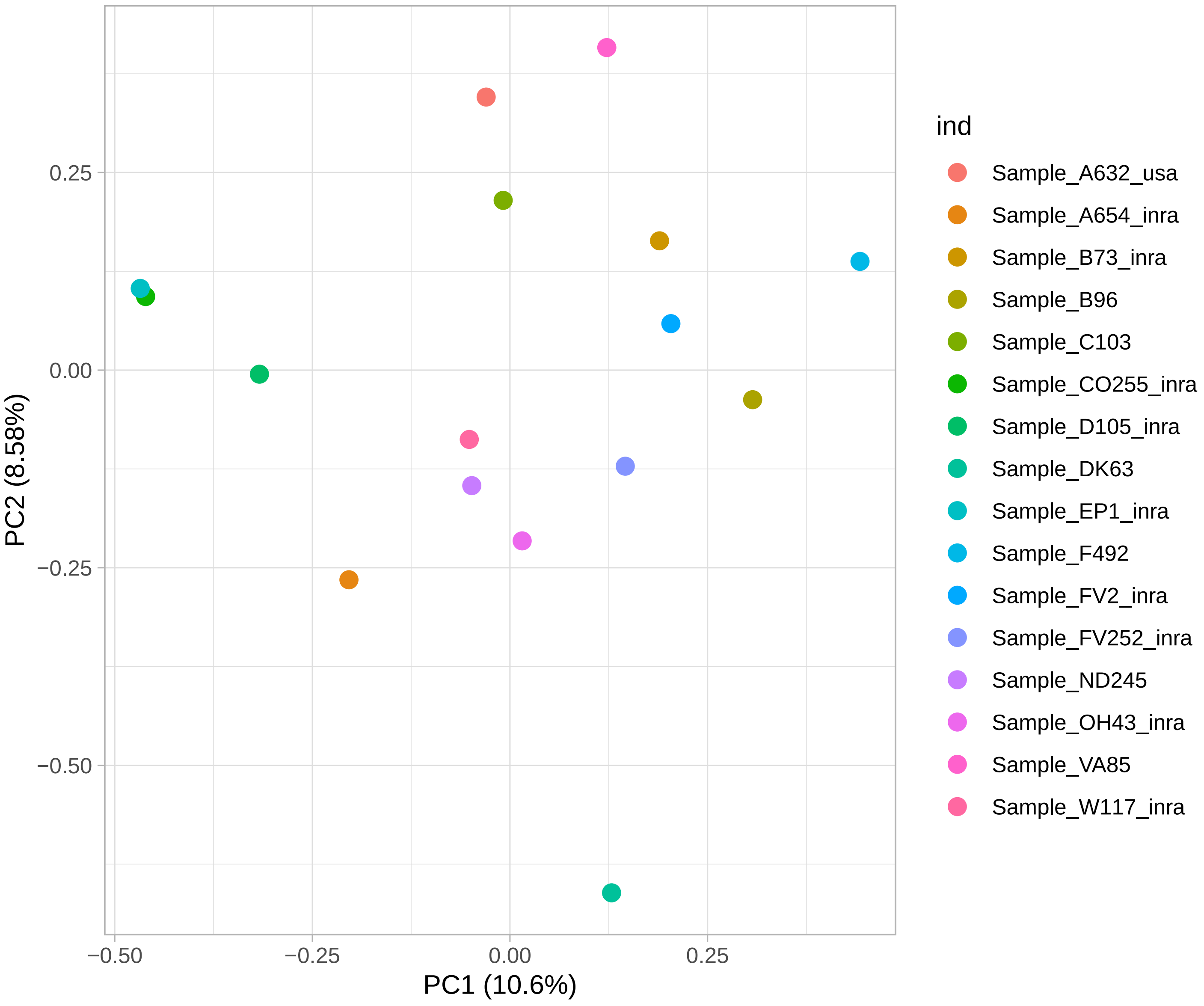

Figure S5 Each founder plotted on the first and second principal components from a principal component analysis of the SNPs within the plasticity GWAS peak for ASI.

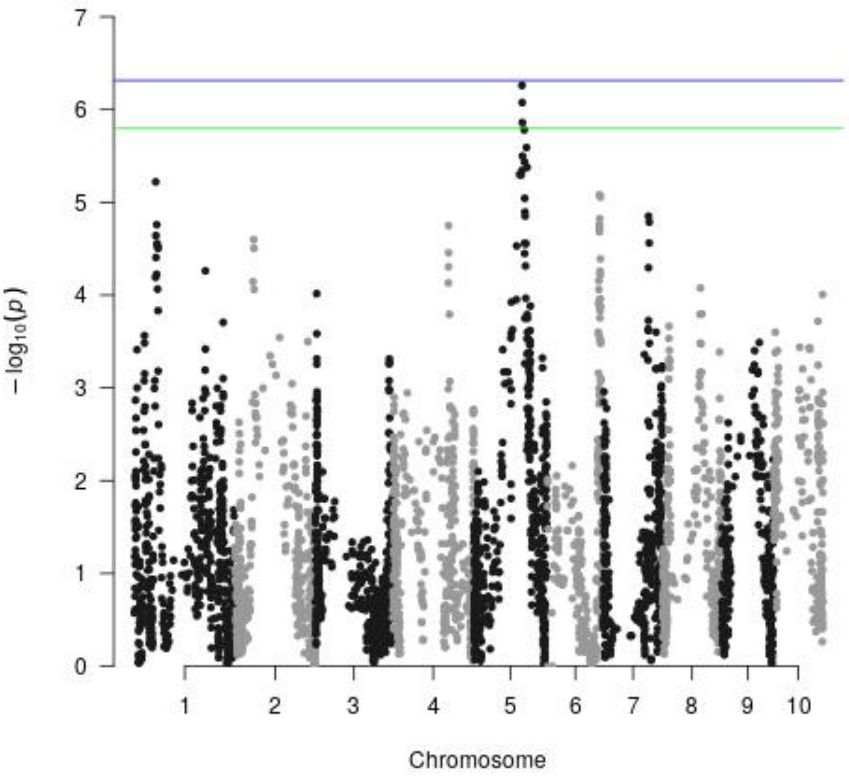

Figure S6 Manhattan plot for plasticity GWAS on grain yield. The blue and green lines represent the 5% and 10% significance levels based on permutation tests, respectively.

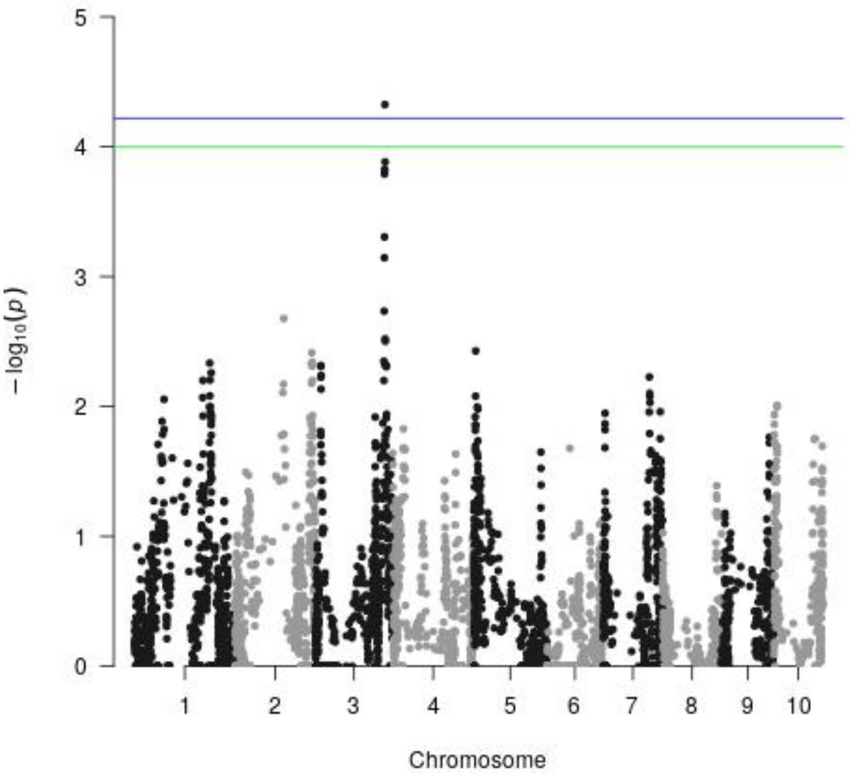

Figure S7 Manhattan plot for Finlay-Wilkinson GWAS on ASI. The blue and green lines represent the 5% and 10% significance levels based on permutation tests, respectively.

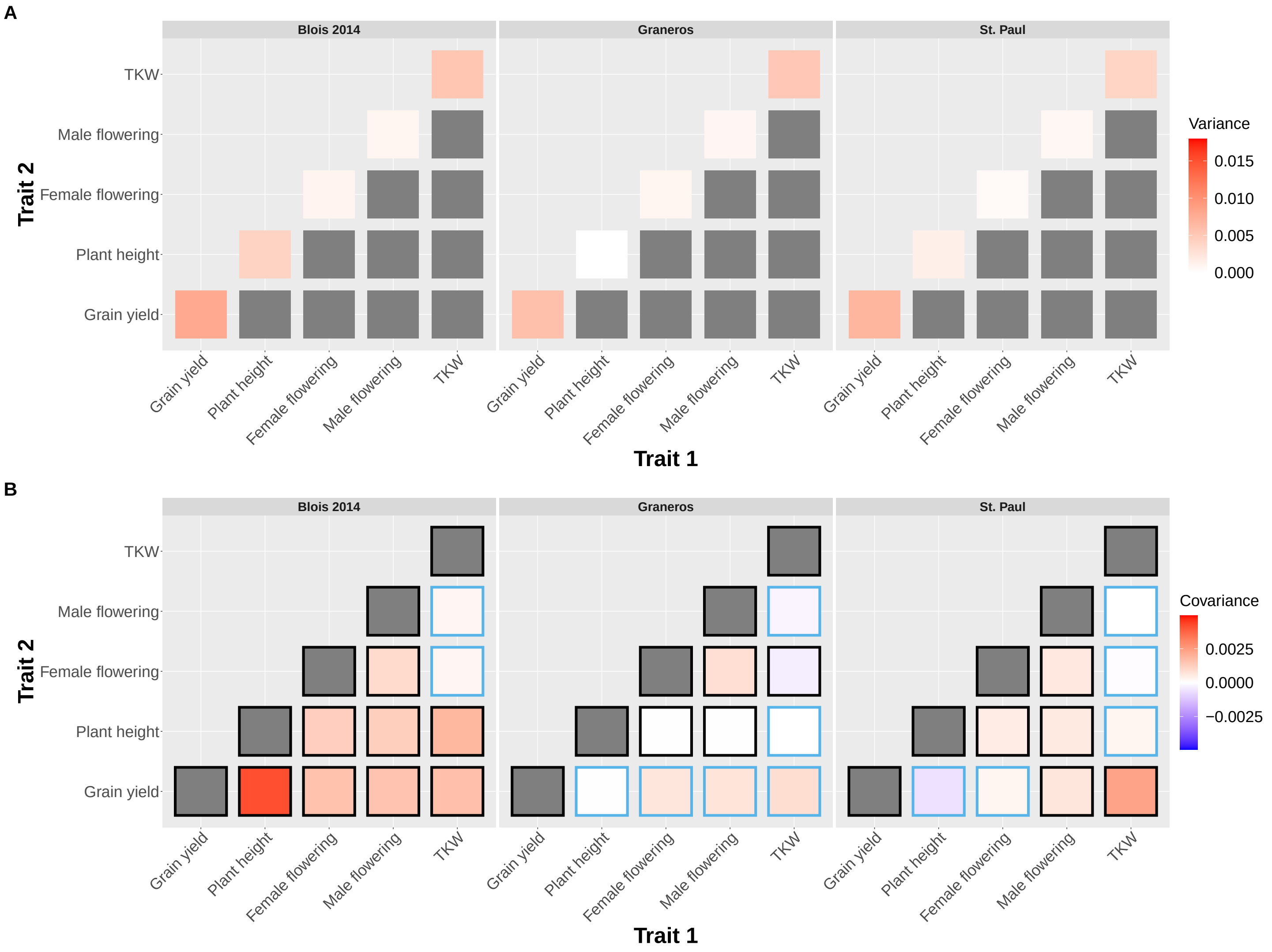

Figure S8 Heat maps of the G-matrices for the remaining environments. Genetic variances are on the top row (A) and covariances are on the bottom row (B). A black border around a covariance indicates that the 95% quantile interval of the posterior does not overlap with zero.

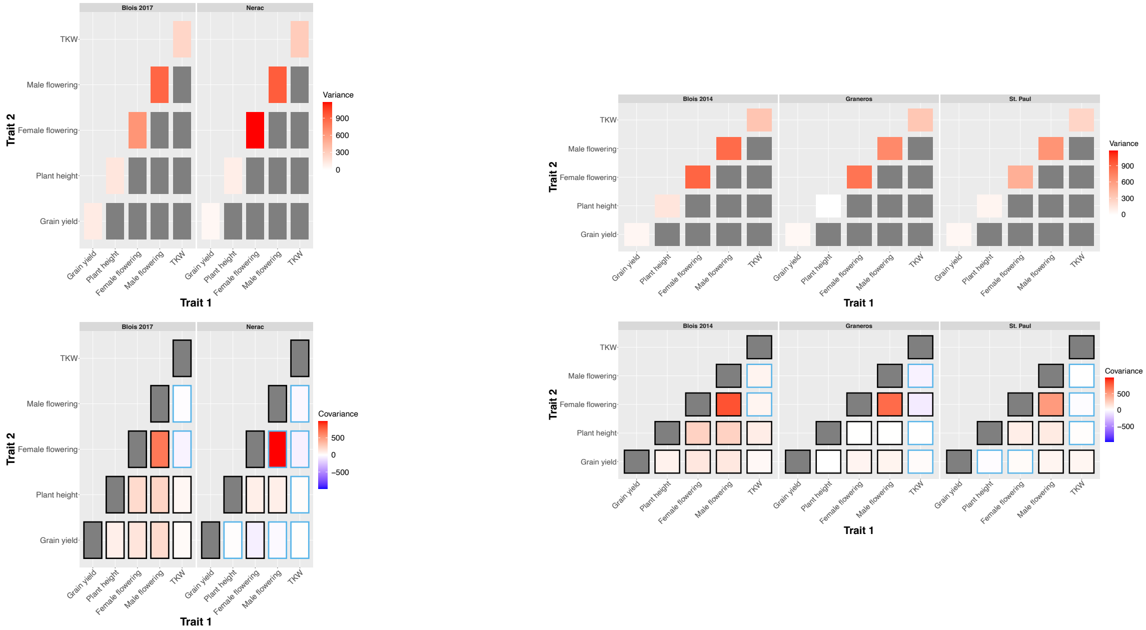

Figure S9 Heat maps of the G-matrices with phenotypes not scaled. Note that this means all traits are on different units. Grain yield is in quintals/ha, plant height is in centimeters, female flowering and male flowering are in GDD, and TKW is is grams. A black border around a covariance indicates that the 95% quantile interval of the posterior does not overlap with zero.

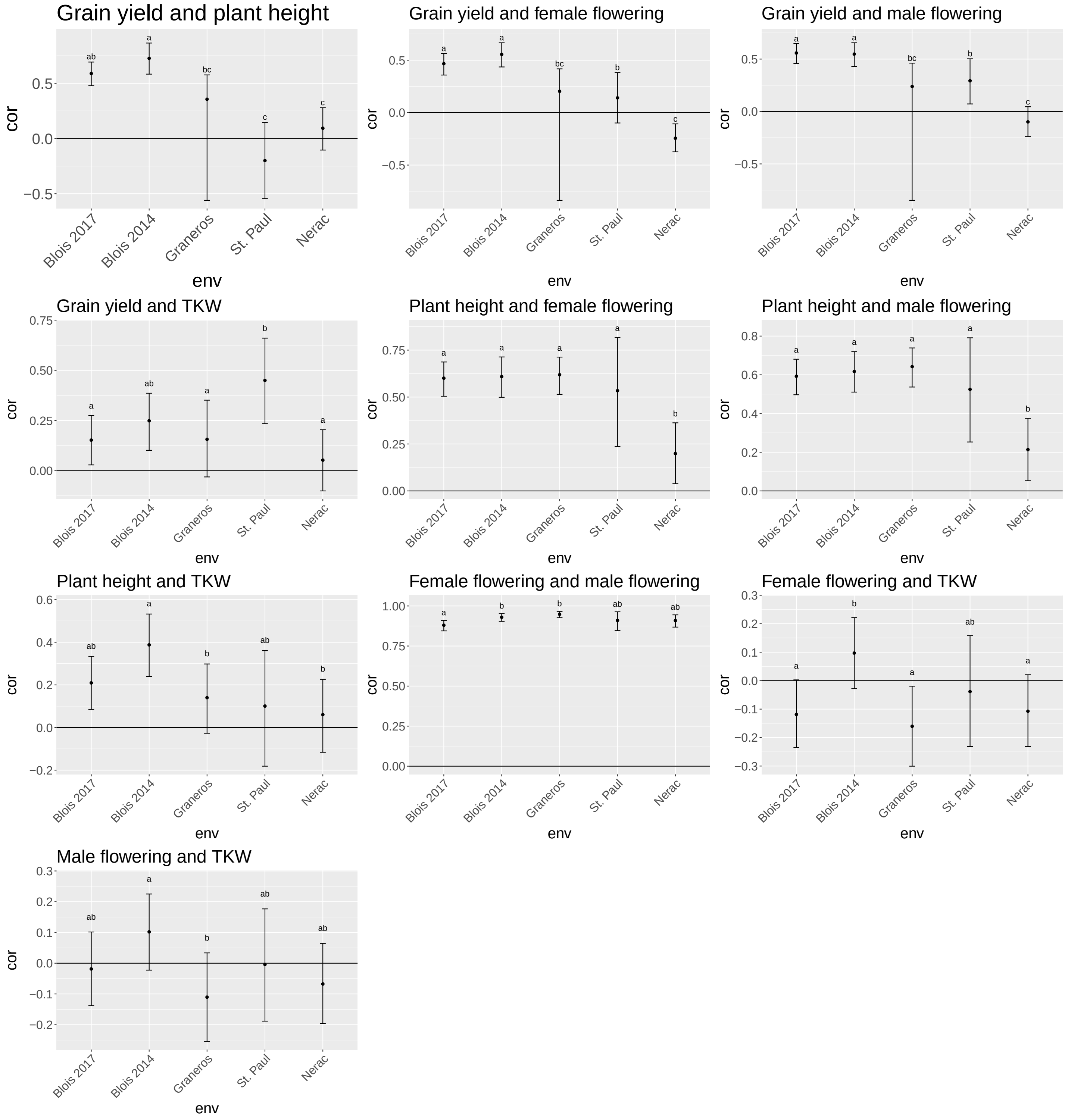

Figure S10 Genetic correlations of each pair of traits. For each pair of traits genetic correlations are shown for each environment with 95% credible intervals. Letters indicate significantly different groups as determined by comparing the 95% credible intervals of the difference between MCMC samples from estimating the correlation in each environment.

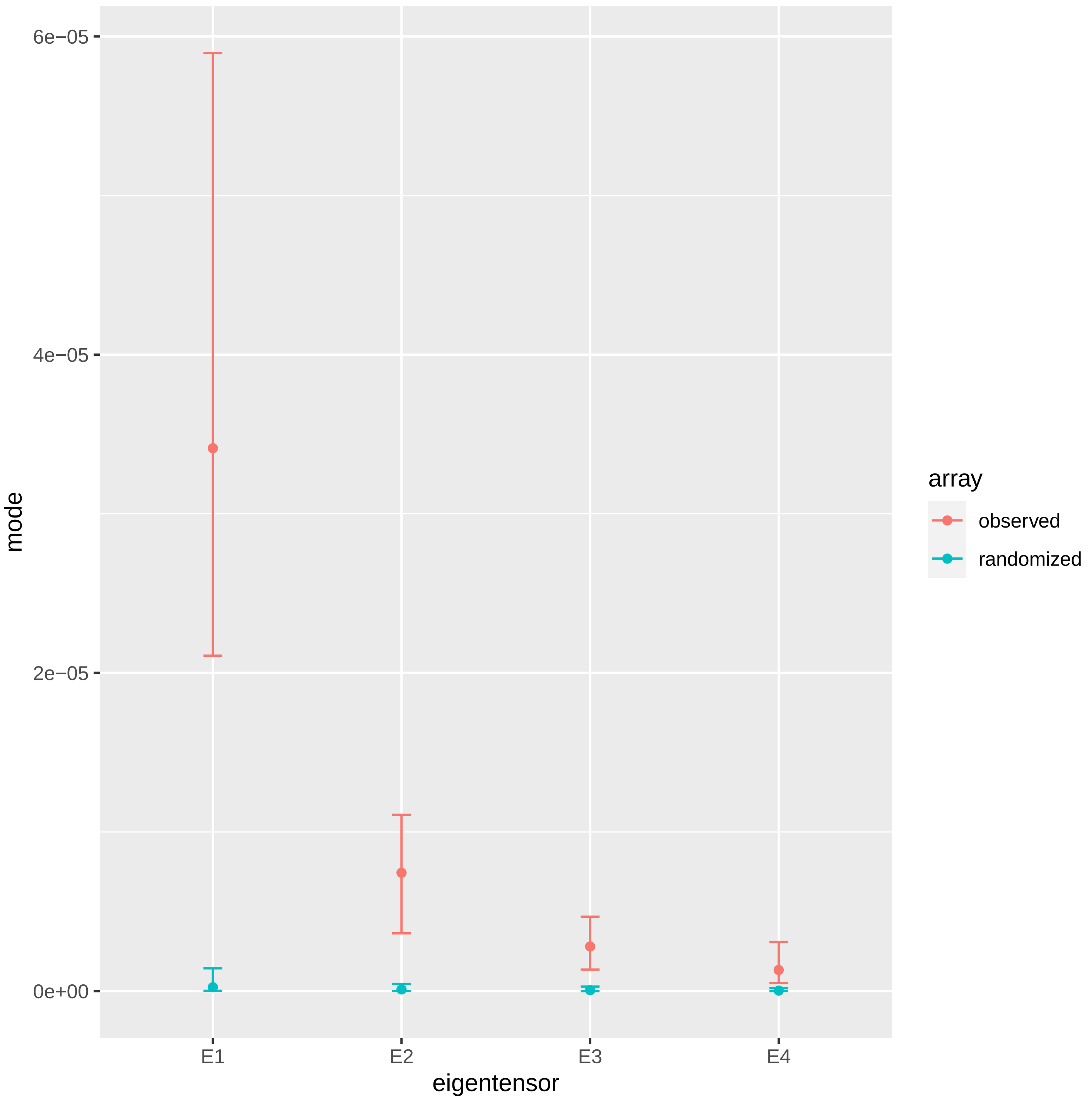

Figure S11 Posterior mode and 95% credible intervals of the eigenvalues of the non-zero eigentensors of the G-matrices. If the observed eigenvalue of an eigentensor is greater than the 95% credible intervals of eigenvalues of the eigentensors estimated from randomized data that indicates the eigentensor explains more of the variation among G-matrices than would be expected by chance.

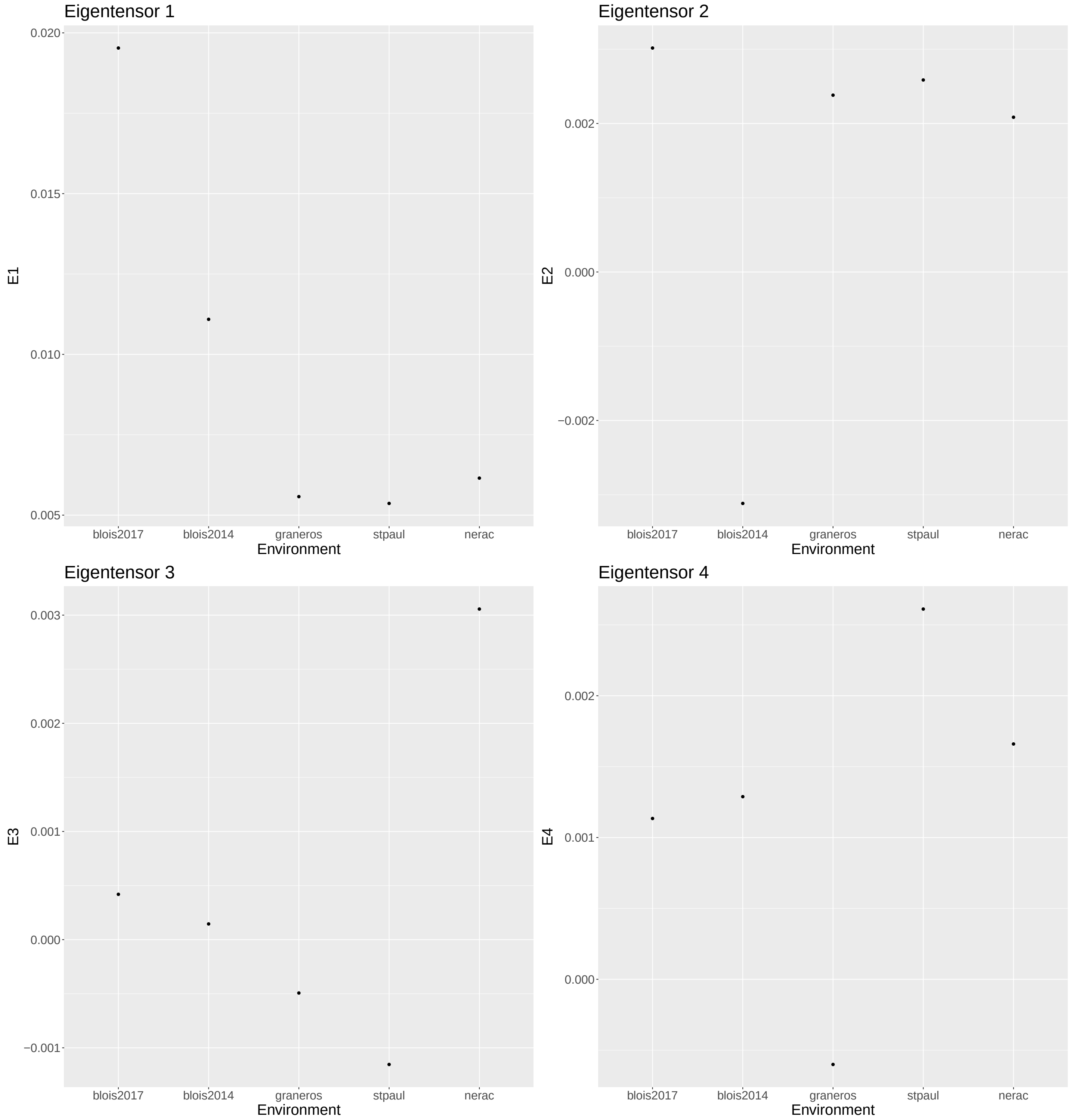

Figure S12 The G-matrix estimated in each environment plotted on each of the four first eigentensors. Note that the scale on the y axis is different for each plot.
